# Development of CSOARG: a single-cell and multi-omics-based machine learning model for ovarian cancer prognosis and drug response prediction

**DOI:** 10.3389/fonc.2025.1592426

**Published:** 2025-05-29

**Authors:** Junyu Chen, Bin Guan, Jihong Zhang, Xin Li, Jingyi Fang, Wencai Guan, Qi Lu, Guoxiong Xu

**Affiliations:** ^1^ Research Center for Clinical Medicine, Jinshan Hospital, Fudan University, Shanghai, China; ^2^ Department of Oncology, Shanghai Medical College, Fudan University, Shanghai, China; ^3^ Department of Obstetrics and Gynecology, Jinshan Hospital, Fudan University, Shanghai, China

**Keywords:** drug sensitivity, gene signature, immunotherapy, ovarian tumor, prognosis, senescence

## Abstract

**Objective:**

Ovarian cancer is the most deadly gynaecological malignancy. This study aims to generate a predictive model for prognosis and therapeutic responses in ovarian cancer using defined specific genes.

**Methods:**

The cellular senescence-associated gene sets and the ovarian aging-associated gene sets from the TCGA and GEO databases were analyzed using Cox regression with LASSO approach and employed to construct a prognostic model of Cellular Senescence and Ovarian Aging-Related Genes (CSOARG). Immunology analysis, functional enrichment, single-cell analysis, and therapeutic responses of ovarian cancer were conducted using the data from public databases. A machine learning model based on the expression levels of prognostic genes combined with clinical features was developed to predict the five-year overall survival. Patients with high- and low-risk scores were separated by the median risk score. Defined genes were verified by qRT-PCR and Western blot. The cellular behavior was evaluated by CCK-8, migration, and wound-healing assays.

**Results:**

After a series of calculations, an 8-gene CSOARG model was generated. CSOARG was correlated with genomic instability that harbored homologous recombination deficiency. The area under the curve (AUC) for 5-year overall survival was 0.68. Patients in the high-risk score group had a higher IC_50_ of chemotherapeutic and targeted therapeutical agents, worse responses to chemotherapy and immunotherapy, and exhibited a poor prognosis. A hub gene *WNK1* was validated and acted as an oncogene affecting ovarian cancer cell viability and migration.

**Conclusions:**

These findings demonstrate that a novel CSOARG model can effectively predict the prognosis and therapeutical responses of patients with ovarian cancer, which may assist clinicians in implementing better practices.

## Introduction

Ovarian cancer is the most lethal gynecological malignancy because of recurrence and drug resistance that leads to a poorer prognosis (1). Ovarian cancer is a highly heterogeneous disease. It is of particular importance to have a predictive method to know the potential treatment and prognosis. Cellular senescence is a state of terminal growth arrest with no cell proliferation (2). Ovarian aging, also known as ovarian failure, is caused by a decline in the number and quality of oocytes and is a physiological process in the alteration of endogenous estrogen hormones (3). Menopause, an important risk factor of ovarian cancer, is closely related to ovarian aging. Furthermore, the incidence of ovarian cancer continues to increase after menopause even beyond a family history of ovarian cancer (4).

It has been shown that many factors influence the prognosis of ovarian cancer patients. Among these, age is the most influential factor that is correlated to clinical phenomena such as tumors. Aging can make the body more susceptible to tumor growth by decreasing immunocompetence and increasing the inflammatory response (5, 6). Cellular senescence has been demonstrated to be associated with antitumor drug resistance because chemotherapy can trigger tumor cell senescence and growth suppression, e.g., adriamycin activates senescence pathways in lung cancer (7). However, the process of cellular senescence may paradoxically promote chemoresistance via the secretion of factors associated with the senescence-associated secretory phenotype (SASP) in colorectal cancer and melanoma (8). Furthermore, radiotherapy has been shown to induce senescence through ionizing radiation-activated pathways in hepatocellular carcinoma (9). The combination of senescence inducers (e.g., PARP inhibitors in breast cancer) with radiotherapy has been shown to amplify therapeutic efficacy (10).

A multitude of prognostic models such as models related to necroptosis, cellular pyroptosis, and cuproptosis have been developed to predict the prognosis of ovarian cancer (11–13). However, no predictive model exists for cellular senescence and ovarian aging with ovarian cancer prognosis. In this study, we developed a predictive model called a CSOARG (Cellular Senescence and Ovarian Aging-Related Genes) for the prognosis of ovarian cancer, which is related to cellular senescence and ovarian aging associated with therapeutical responses, and validated the effect of a hub gene *WNK1* on ovarian cancer cell viability and migration.

## Materials and methods

### Data sources

The Cancer Genome Atlas Ovarian Cancer (TCGA-OV) dataset was downloaded from the UCSC Xena Browser (https://xenabrowser.net/) (14). After samples without clinical information about age, stage, or overall survival time of less than 30 days were removed, 407 tumor samples were included for further analysis. The single-cell RNA sequencing (scRNA-seq) dataset GSE165897 was collected from Gene Expression Omnibus (GEO) (https://www.ncbi.nlm.nih.gov/geo/) (15). For external validation, Bulk RNA sequencing (Bulk RNA-seq) datasets GSE140082 and GSE30161 were downloaded from GEO by the “GEOquery” R package.

### Prognostic cellular senescence and ovarian aging-related genes

The workflow of this study is presented in **Supplementary Figure S1**. Two datasets were selected to analyze and generate a model. A total of 314 ovarian aging-related genes were selected from a previous study (16) (**Supplementary Table S1**). A total of 125 genes referred to cellular senescence genes (**SupplementaryTable S2**) were obtained from the SenMayo geneset (17). The ovarian aging-related genes and the SenMayo geneset genes were merged to generate a predictive model of Cellular Senescence and Ovarian Aging-Related Genes (CSOARG). Prognosis-related genes were screened for the construction of prognostic models. Bulk RNA-seq and scRNA-seq analyses were performed on the predictive function of the models. Clinical samples collected at the Jinshan Hospital of Fudan University were used to analyze the relationship between gene expression in the model and clinical characteristics. Then, a hub gene *WNK1* was screened and validated *in vitro* at the cellular level.

### Establishment of CSOASRG signature in ovarian cancer

Univariate Cox Regression analysis was applied to screen prognostic genes from CSOARG in the TCGA-OV dataset. Then, LASSO Cox Regression analysis was performed to construct a prognostic CSOARG model through the “glmnet” R package. The risk score for each cell in the single-cell data was calculated and the formula that calculated the risk score was as follows: Risk score=∑coef(gene i)*expression(gene i). The threshold used for the separation between high- and low-risk groups was taken by the median of all cell sub-risk scores. The coef is listed in **Supplementary Table S3**. Time-dependent receiver operating characteristic (ROC) analysis was performed to evaluate the efficiency of the model by the “time ROC” R package.

Patients in the TCGA-OV dataset were then divided into two groups by a mean risk score: high-risk group, and low-risk group. The overall survival (OS) time between the two groups was compared by Kaplan-Meier analysis and demonstrated by Kaplan-Meier plot using the “survival” and “survminer” R packages. The “forestplot” package was applied to draw the forestplot. A nomogram was constructed to predict a 5-year OS probability and the calibration curve was performed to test the precision of this nomogram using the “rms” R package. Kyoto Encyclopedia of Genes and Genomes (KEGG) and Gene Ontology (GO) analyses were employed to demonstrate functional classification and related pathways of prognostic CSOARG using “org.Hs.eg.db” and “clusterProfiler” R packages (18).

### Immunologic function analysis

Single sample gene setenrichment analysis (ssGSEA) algorithm was applied to estimate the expression of model genes in 29 immune cell subsets (19) by the”GSVA” R package (20). CIBERSORT (https://cibersort.stanford.edu/) was devoted to profiling the relative proportion of 22 subsets of immune cells between high- and low-risk groups (21). Differential expression between two risk groups of 79 immune checkpoint genes from published papers (22–24) was determined by the “limma” R package.

### Immunotherapeutic responses prediction

The immunophenoscores (IPS) of ovarian cancer were downloaded from The Cancer Immunome Atlas (TCIA) database (https://tcia.at/) (25). IPS were compared between high- and low-risk groups. Tumor Immune Dysfunction and Exclusion (TIDE) scores were calculated by the TIDE database website (http://tide.dfci.harvard.edu) (26).

### Drug sensitivity prediction

The half-maximal inhibitory concentrations (IC_50_) which represented drug sensitivity were predicted by the “pRRophetic” package (27). Differences in predicted drug sensitivity were compared between high- and low-risk groups in the TCGA-OV dataset.

### Genomic instability analysis

The data on tumor mutation burden (TMB), homologous recombination deficiency (HRD) scores, somatic alteration, and single nucleotide variants (SNV) in ovarian cancer patients were downloaded from the TCGA database. TMB and HRD scores were compared between high- and low-risk groups. The **“**maftools**”** R package was used to analyze and visualize somatic alteration and SNV in two risk groups.

### Single-cell RNA-seq analysis

TME was analyzed based on the dataset of GSE165897 (28). The **“**Seurat**”** R package was applied to run cell quality control and normalize the gene expression in each cell with default parameters (29). Each cell was defined by cell definition in this dataset and cell clusters were displayed by T-Distributed Stochastic Neighbor Embedding (t-SNE) algorithms. The communication signaling network between tumor and plasma cells was examined by the **“**NicheNETr**”** R package (30).

### Machine learning

A prognostic model was constructed by Python software and the “PyCaret” package in the method of machine learning in the TCGA-OV database. The model was developed by the features of age, FIGO (International Federation of Gynecology and Obstetrics) stage, and the expression of 8 prognostic genes. Stratified ten-fold cross-validation was performed and 15 classification algorithms were compared to find the best model for predicting the prognosis of ovarian cancer patients using the “PyCaret” Python package (https://pycaret.org/).

### Clinical tissue sample collection

Ovarian cancer tissues (n=16) and non-cancerous ovarian tissues (para-cancerous tissues or non-tumor tissues; n=24) were collected from Jinshan Hospital of Fudan University after surgery and stored with liquid nitrogen. Informed consent was obtained from each patient and the ethical approval of the study for human subjects was approved by the Ethics Committee of Jinshan Hospital (approval no. JIEC-2023-S68). All methods were performed in accordance with the relevant guidelines and regulations.

### Cell culture

The human immortalized ovarian surface epithelial cell lines IOSE-80 (derived from normal ovarian surface epithelium; OriGene Technologies, Inc. Wuxi, Jiangsu, China), human ovarian cancer cell lines A2780 (derived from ovarian endometrioid adenocarcinoma) and SK-OV-3 (derived from ovarian endometrioid adenocarcinoma in ascites) were obtained from American Type Culture Collection (ATCC), VA, USA and cultured in DMEM with 10% fetal bovine serum (FBS; Thermo Fisher Scientific, Inc, MA, USA). The human ovarian cancer cell line OVCAR-3 (derived from ovarian adenocarcinoma in ascites; ATCC) was cultured in RPMI-1640 medium (Thermo Fisher) with 20% FBS. All cells were maintained in a humidified incubator at 37°C and 5% CO_2_, and their identity was confirmed by short tandem repeat (STR) analysis. They were also routinely tested for the free of pathogens and mycoplasma.

### RNA extraction and quantitative real-time reverse transcription-PCR

Total RNA from tissues and cells was extracted using an RNA-Quick Purification Kit (Shanghai Yishan Biotechnology Co. Ltd, Shanghai, China) and was reverse-transcribed to cDNA using a qPCR RT kit (Roche Diagnostics, IN USA). cDNA was amplified by BeyoFast SYBR Green qPCR Mix (2X; High ROX; Beyotime, Shanghai, China). The primer sequences are listed in **Supplementary Table S4**.

### Extraction of proteins and western blotting

The extraction of total proteins was conducted using the Minute™ SD-001 kit (Invent Biotechnologies, Inc, Beijing, China), supplemented with 1% phenylmethanesulfonyl fluoride (Beyotime) and 1% phosphatase inhibitor (Nanjing KeyGen Biotech Co, Ltd, Nanjing, Jiangsu, China). The 7.5% SDS-PAGE was used to separate proteins which were then transferred to a 0.45 mm PVDF membrane. A transfer buffer containing 10% methanol and 0.1% SDS was prepared by diluting a 10X stock solution in distilled water. Proteins were transferred under a constant current of 300 mA for 2 h. Primary antibodies were rabbit anti-WNK1 (1:500 dilution, Cat No: 28357-1-AP) and rabbit anti-Vinculin (1:5000 dilution, Cat No: 28357-1-AP) purchased from Proteintech lnc. (Wuhan, Hubei, China). These antibodies were incubated overnight at 4^0^C. The secondary antibody was horseradish peroxidase-conjugated goat anti-rabbit IgG (1:10000 dilution; Proteintech). Signals were detected using a BeyoECL Moon kit (Beyotime), followed by quantification utilizing ImageJ software.

### Detection of cell viability and migration

For cell viability, cells were transfected with WNK1-siRA (si-WNK1) first and re-cultured in 96-well plates (6000 cells per well in A2780 and 8000 cells per well in OVCAR-3). Non-specific siRNA was used as a negative control (NC). Cell viability was assessed using a Cell Counting Kit-8 kit (CCK-8; Yeasen Biotechnology, Shanghai, China) at 0, 24, 48, and 72 h, respectively.

For the transwell assays, A2780 and OVCAR-3 cells were transfected with si-WNK1 (sequence: 5**’**-GCGACGACUACGAGAUAAATT-3**’**) or NC siRNA (sequence: 5**’**-UUCUCCGAACGUGUCACGUTT-3**’**) for 2 days. After harvest by centrifugation and resuspension with serum-free medium, cells were plated into the upper chamber of Transwell plates (Corning Incorporated, Corning, NY, USA). At the same time, a complete medium supplemented with 20% FBS was added to the lower chamber of the Transwell plate. After incubation for 48 h, cells located on the bottom surface of Transwell were fixed with 4% paraformaldehyde for 15 min and stained with crystal violet for 30 min. Migrating cells were photographed in three random fields of view using an inverted microscope (Olympus, Tokyo, Japan) and counted using ImageJ (National Institutes of Health, Bethesda, USA).

For the wound healing assays, siRNA-transfected A2780 and OVCAR-3 cells were seeded in 6-well plates and scraped with a 200 μL pipette tip when 90% confluence was reached. The scratch width was measured at 0 and 48 h after scratching. The percentage of wound healing was calculated using the following formula: (original scratch width after 48 h culture)/(original scratch width) × 100%.

### Statistical analysis

All statistical analyses were conducted using R software (v4.2.1, R Foundation for Statistical Computing, Vienna, Austria). The Student**’**s *t*-test and Wilcoxon rank sum test were used to analyze the differences between the two groups. One-way analysis of variance (ANOVA), followed by Tukey**’**s multiple comparisons test, was used for multiple group comparisons. Data were presented as the mean ± standard deviation (SD). A P-value < 0.05 was defined as statistical significance.

## Results

### Construction of the predictive model of prognosis in ovarian cancer

**Figure 1:**
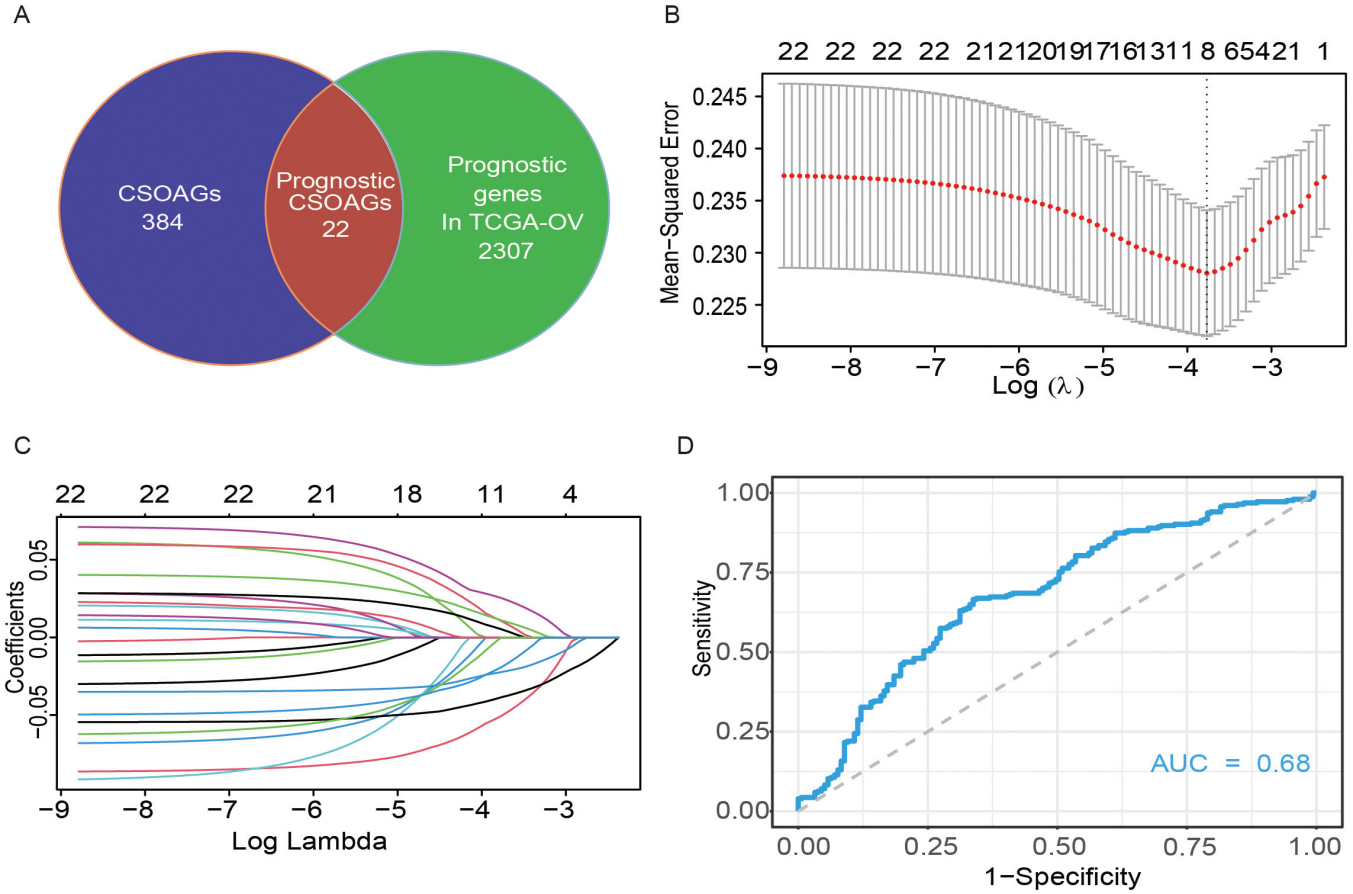
Construction of a predictive model of CSOARG for prognosis in ovarian cancer. **(A)** Screening of genes related to the prognosis of cellular senescence and ovarian aging. **(B)** Selection of the optimal candidate genes in the LASSO model. **(C)** LASSO coefficients of prognosis-associated CSOARG. Each curve represents a gene. **(D)** The time-dependent ROC curve at 5-year OS in the TCGA-OV cohort.

A total of 406 genes from datasets related to cellular senescence and ovarian aging were obtained from the literature (16, 17). We name these genes as cellular senescence and ovarian aging-related genes. Further, a total of 2329 prognosis-related genes were screened from the TCGA database by the Unicox regression with a threshold of P <0.05. A set of 22 prognostic senescence- and tumor senescence-related genes were obtained (**Figure 1A**). These 22 genes were further integrated into the LASSO regression analysis and validated by 10-fold cross-validation (**Figures 1B, C**). Thus, an 8-gene model was obtained (see components of the model in **Supplementary Table S3**). Time-dependent ROC curves were used to evaluate the efficiency of the model. The area under the curve (AUC) of the model for 5-year overall survival was 0.68 (**Figure 1D**).

### Clinical significance of prognostic modeling

**Figure 2:**
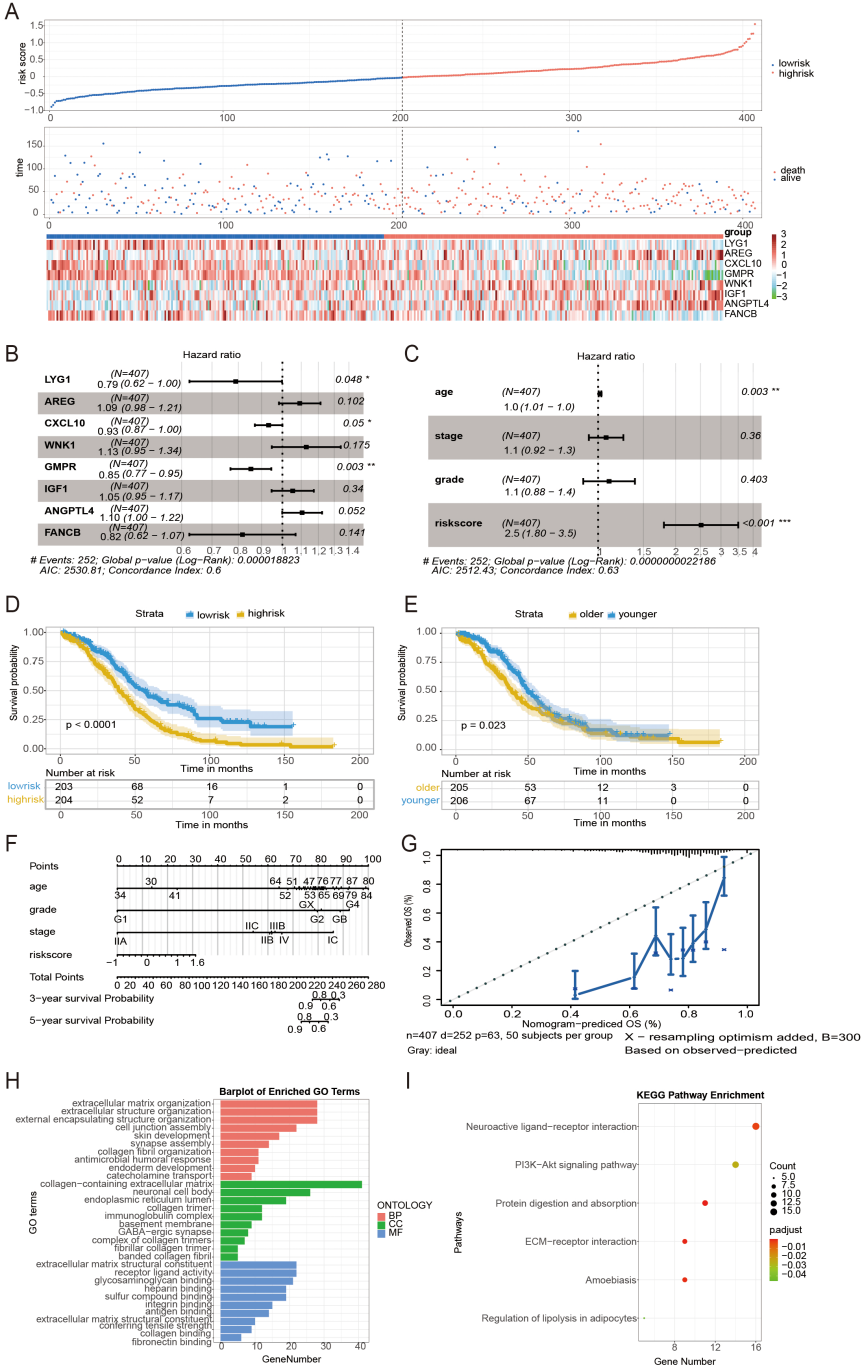
Analysis of the clinical function of predictive models for prognosis. **(A)** Distribution of risk scores in the TCGA and patient distribution in the high- and low-risk groups based on the overall survival (OS) status. The heatmap shows expression profiles of the 8 genes in the CSOARG mode. **(B)** Forest plots showing results of univariate Cox regression analysis between the expression of 8 candidate genes and overall survival. **(C)** Forest plots showing results of univariate Cox regression analysis between 8 genes in the CSOARG model. **(D)** Kaplan-Meier curves for the OS of patients in the high- and low-risk groups. **(E)** Kaplan-Meier curves for the OS of patients in the order and younger groups. **(F)** The nomogram plot integrated CSOARG risk score, age, stage, and grade in the TCGA-OV training cohort. **(G)** The calibration plot for the probability of 5-year OS in the TCGA-OV cohort. **(H)** A bar plot of the Gene Ontology (GO) enrichment analysis of differential genes in high- and low-risk groups. **(I)** A bar plot of the Kyoto Encyclopedia of Genes and Genomes (KEGG) enrichment analysis of differential genes in high- and low-risk score groups. *p<0.05; **p<0.01; ***p<0.001.

Based on the median risk score, samples of patients were separated into two groups, high- and low-risk score groups (**Figure 2A**). Unicox regression analysis was employed to analyze the association between risk score, age, International Federation of Gynecology and Obstetrics (FIGO) stages, and prognosis in patients with ovarian cancer. A substantial body of research had identified a robust correlation between the genes CXCL10, LYG1, and GMPR and patient prognosis (**Figure 2B**). A forestplot showed that the correlation between the risk score and prognosis, as well as between age and prognosis, was found to be statistically significant within the prediction model. (**Figure 2C**). A survival analysis revealed that patients in the high-risk score group exhibited a worse prognosis (**Figure 2D**) and appeared with older age (**Figure 2E**). A nomogram was constructed to predict a 5-year overall survival probability, in which predictive factors included age, tumor stage, and risk score (**Figure 2F**). The calibration curves were used to validate the reliability of the line graph for predicting 5-year survival and resulted in an effective prediction of the 5-year survival rate of ovarian cancer patients (**Figure 2G**). The results of the Gene Ontology (GO) term analysis in the categories of Biological Process, Cellular Component, and Molecular Function indicated that the differential genes between high- and low-risk groups were involved in extracellular matrix organization, collagen-containing extracellular matrix, and extracellular matrix structural constituent (**Figure 2H**). The result of the KEGG enrichment analysis of differential genes in high- and low-risk groups exposed several functional pathways such as neuroactive ligand-receptor interaction, PI3K-Akt signaling pathway, etc (**Figure 2I**). These functional enrichments suggest that the extracellular microenvironment is the primary focus of interest.

### Immune function analysis

**Figure 3:**
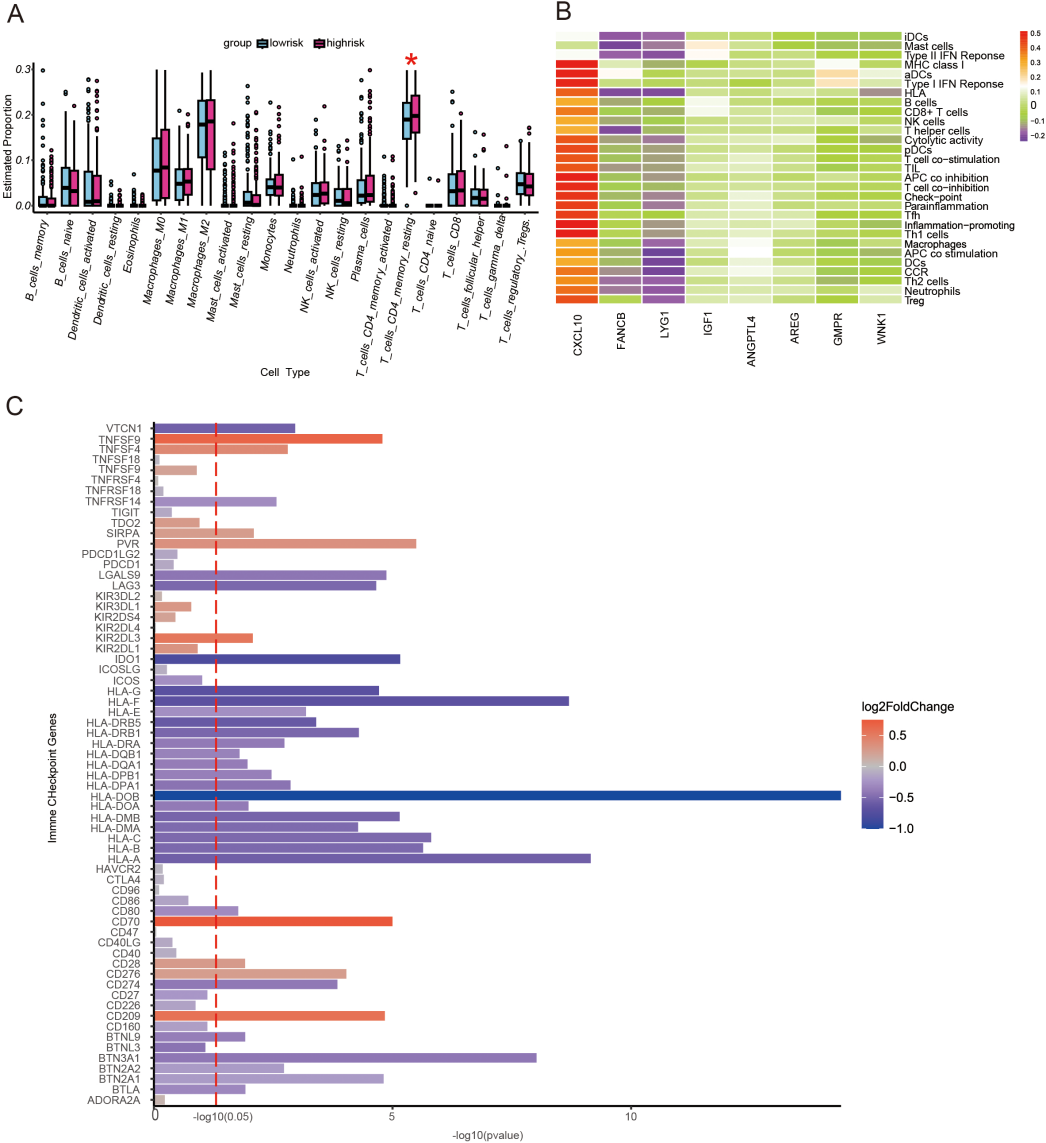
The landscape of immune function and immune cell infiltration between the high- and low-risk groups in the TCGA cohort. **(A)** Boxplot showing differences in immune functions between the high- and low-risk score groups. *P<0.05. **(B)** A heat map of prognostic genes in different types of immune cells. **(C)** A bar plot demonstrating the differential expression of immune checkpoint genes in high- and low-risk score groups.

Two algorithms, CIBERSORT and GSEA, were used to assess immune infiltration status. The CIBERSORT algorithm was used to assess the difference in the proportion of immune cells composed between high- and low-risk groups. The high-risk score group exhibited higher counts of resting memory CD4 T cells (**Figure 3A**). The GSEA algorithm was used to assess the expression levels of genes in immune cells in the predictive model of prognosis. We found that CXCL10 expression was significantly higher in various immune cells (**Figure 3B**). Furthermore, there were 79 immune checkpoint-related genes collected from the literature to be differentially expressed between high- and low-risk score groups (**Figure 3C**). Most of the immune checkpoint genes were down-regulated in the high-risk group, especially the HLA molecular family. These data indicate that a weaker immune function and less effective immunotherapy in the high-risk score group may exist.

### Prediction of effects of immunotherapy

**Figure 4:**
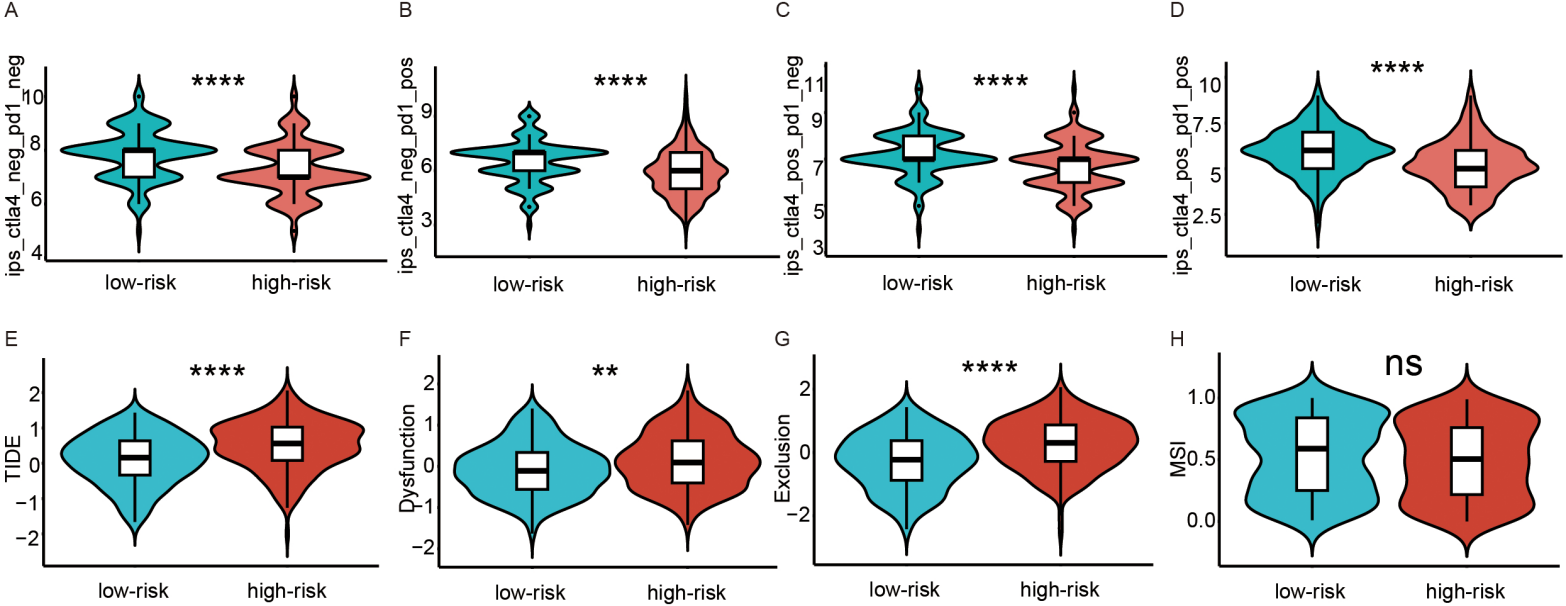
Analysis of immunotherapy response in high and low-risk groups. **(A–D)** Comparison of immunophenoscores (IPS) between the low- and high-risk score groups. **(A)** PD-1 and CTLA-4 were both negatively expressed. **(B)** only PD-1 was positively expressed. **(C)** only CTLA-4 was positively expressed. **(D)** PD-1 and CTLA-4 were both positively expressed. **(E–H)** Comparison of tumor microenvironment scores calculated by TIDE between the high- and low-risk score groups. **(E)** Total TIDE scores. **(F)** T-cell dysfunction scores. **(G)** T-cell excretion scores. **(H)** Microsatellite instability scores. ns, not significance; **p<0.01; ****p<0.0001.

To reflect the differential expression of the immune checkpoint molecules PD-1 and CTL-4, IPS scores in high- and low-risk score groups were compared. We found that patients in the low-risk score group had higher levels of PD-1 and CTL-4 expression (**Figures 4A–D**), suggesting better immune efficacy in low-risk models. Next, TIDE scores were utilized to analyze variations in immune cell function between high and low-risk score groups. We found that TIDE scores, immune cell T-cell exclusion, and functional dysfunction were higher in the high-risk score group compared to the low-risk score group (**Figures 4E–D**). Additionally, there were no significant differences in microsatellite instability between these 2 groups. Furthermore, T-cell-associated cellular immunity was lower in the high-risk score group. However, there was no significant difference in the level of tumor cell-specific antigen exposure due to microsatellite instability between the high- and low-risk score groups.

### Correlation between CSOARG and genomic instability

Patients with low-risk scores of CSOARG had more mutation burden and HRD than those with high-risk scores in the TCGA-OV cohort (**Supplementary Figures S2A, B**). However, no difference in gene mutations was found between the subtypes of the high- and low-risk scores (**Supplementary Figures S2C–F**).

### Drug sensitivity prediction

**Figure 5:**
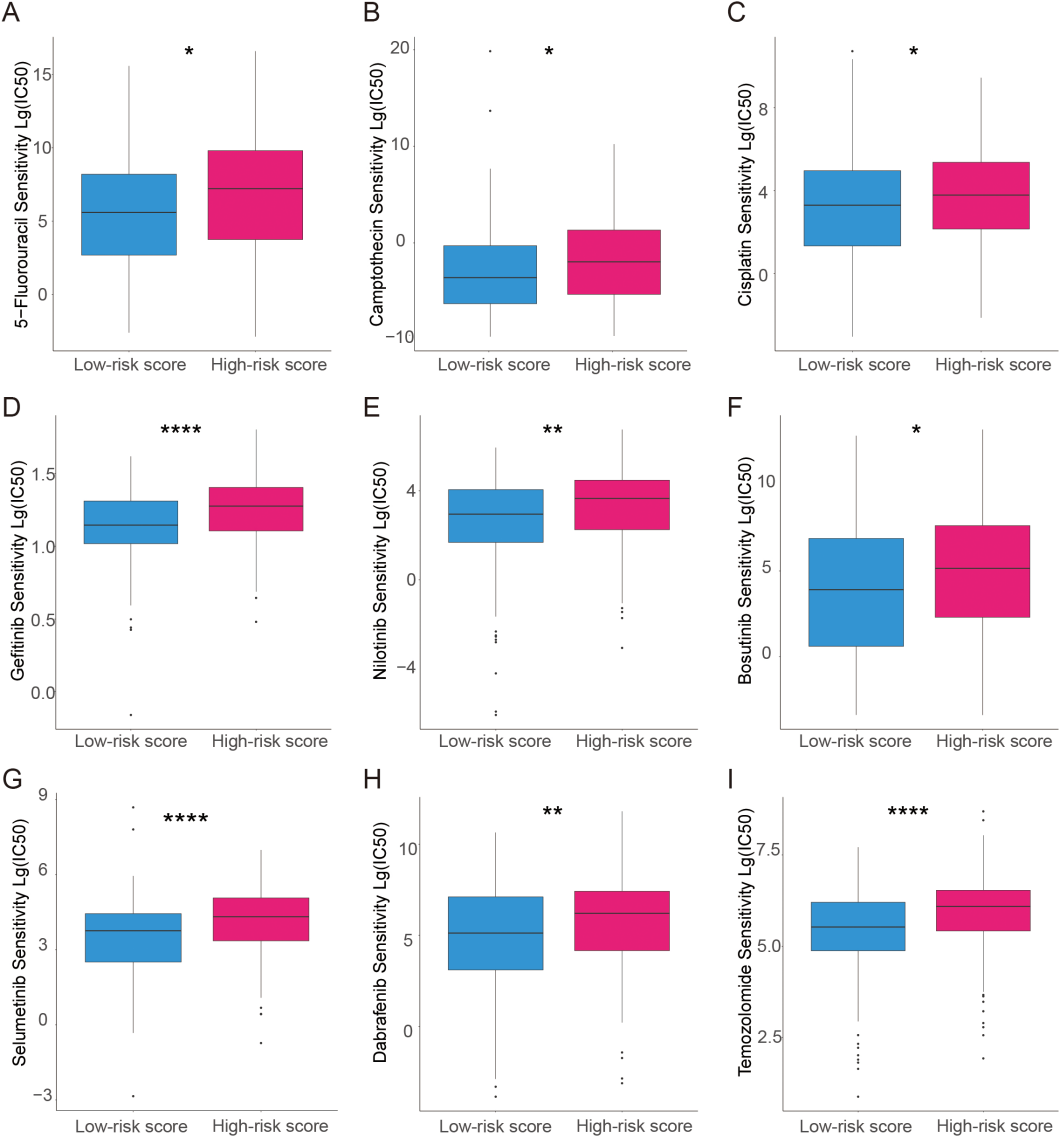
Analysis of the association between the risk model and chemotherapeutics, endocrine therapy, and targeted therapy. **(A–C)** The model predicts the sensitivity to chemosensitivity. It was estimated that patients with low-risk scores had lower IC_50_ for chemotherapeutics of 5-fluorouracil, Camptothecin, and Cisplatin. **(D–H)** The model predicts the sensitivity to targeted therapy. It was estimated that patients with low-risk scores had lower IC_50_ of Gefitinib, Nilotinib, Bosutinib, selumetinib, and Dabrafenib. **(I)** The model predicts the sensitivity to endocrine therapy. It was estimated that patients with low-risk scores had lower IC_50_ of Temozolomide. *p<0.05; **p<0.01; ****p<0.0001.

The relationship between prognostic modeling and the efficacy of chemotherapy or targeted agents was further explored. For each patient in the TCGA dataset, the pRRophetic package was used to predict drug IC_50_ values. Patients in the high-risk score group had a higher IC_50_ of chemotherapeutic agents such as cisplatin, 5-FU, and camptothecin (**Figures 5A–C**). Patients in the high-risk group also had a higher IC_50_ for targeted therapeutics such as Gefitinib et al. (**Figures 5D–H**) and an endocrine agent such as Temozolomide (**Figure 5I**). These data suggest that high-risk scores are associated with worse chemotherapy and targeted therapy.

### Single-cell analysis

**Figure 6:**
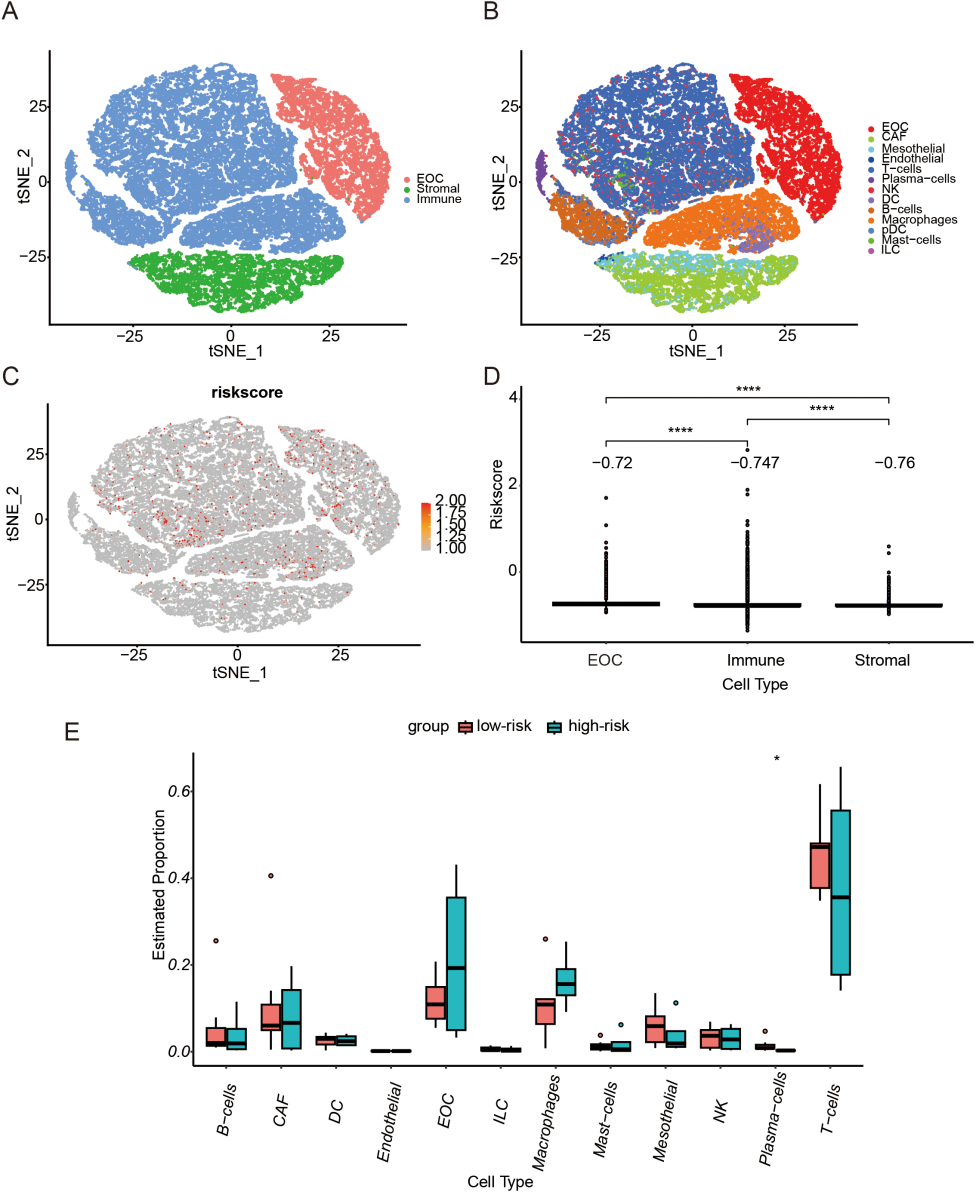
Single-cell RNA-seq Analysis. **(A)** T-distributed Stochastic Neighbor Embedding (t-SNE) representation of single cells color-coded by major cell type: Malignant (EOC, Epithelial ovarian cancer), Stromal, and Immune. **(B)** t-SNE representation of single cells. **(C)** t-SNE plot showing the CSOARG risk scores of whole tissue cells. **(D)** Bar plot showing differences in CSOARG risk scores by cell types. ****p<0.0001. **(E)** Bar plot showing differences between high- and low-risk scores of CSOARG by subcell type. *p<0.05.

**Figure 7:**
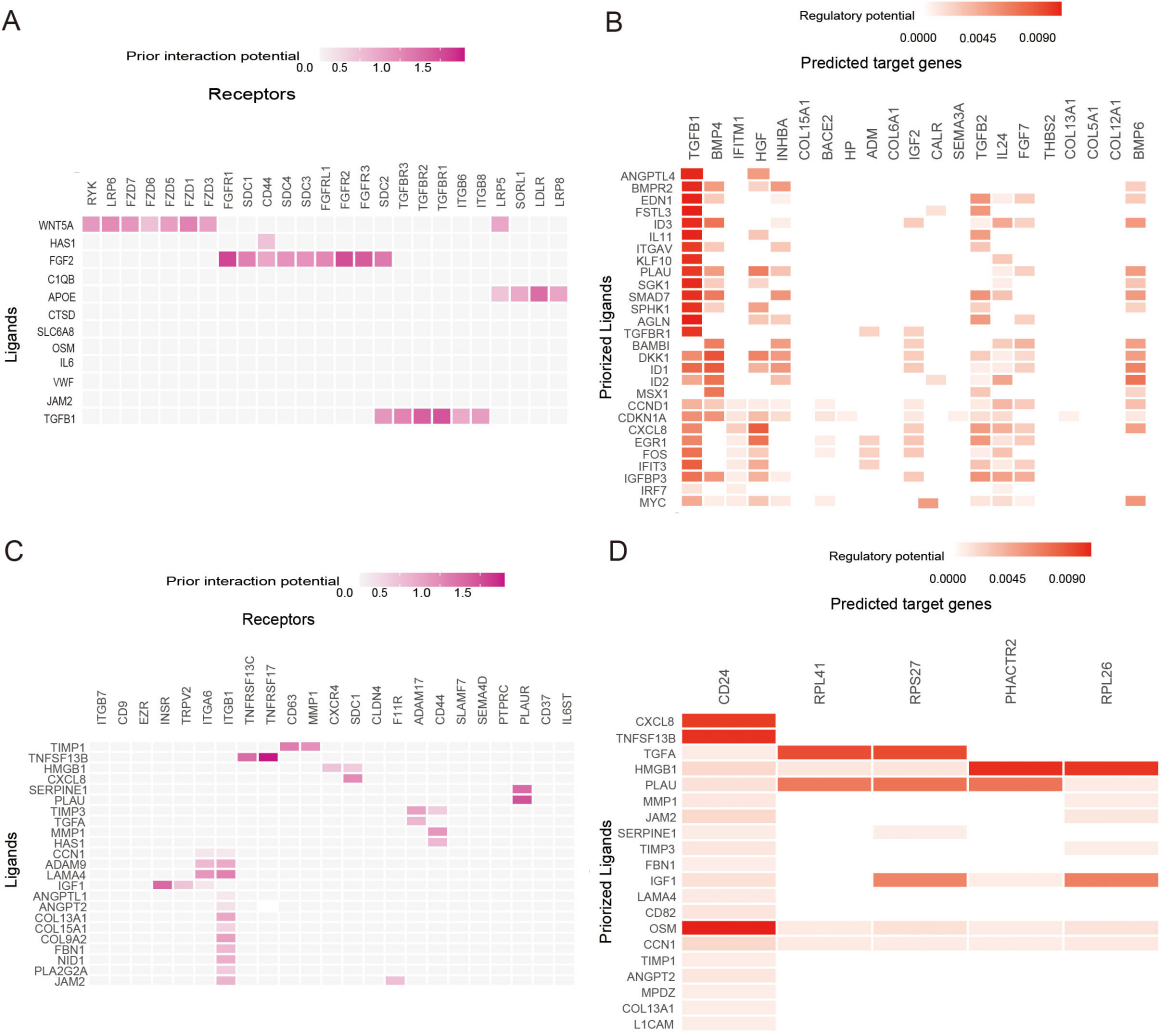
Ligand−Receptor Analysis. **(A)** Heatmap shows the interactions of the prioritized ligand in plasma cells with the receptors expressed in epithelial ovarian cancer (EOC) cells. **(B)** Heatmap shows the regulatory potential of the prioritized ligands in plasma cells that drive the cellular state of EOC cells. **(C)** Heatmap showing the interactions of the prioritized ligand in EOC cells with the receptors expressed in plasma cells. **(D)** Heatmaps show the regulatory potential of the prioritized ligands in EOC cells that drive the cellular state of plasma cells.

Based on the annotations in the published literature (28), different cell types and sub-cell types, including epithelial ovarian cancer (EOC), stromal cells, and immune cells that were further divided into sub-cell types such as CAF cells and NK cells, were distinguished (**Figures 6A, B**). Additionally, the risk scores for each cell were distributed (**Figure 6C**). Among the three cell types, tumor cells had the highest risk score, followed by immune cells, whereas stromal cells had the lowest risk score (**Figure 6D**). Differences in the proportions of subcellular composition between high- and low-risk score tissues were compared. The percentage of plasma cells was significantly lower in patients with the high-risk score (**Figure 6E**). Ligand/receptor analysis was used to analyze cellular communication between tumor cells and plasma cells and their tumor microenvironment. Firstly, tumor cells were analyzed using receptors as markers. Tumor cells were regulated by secreted TGF-β from the surrounding environment. Several molecules such as CXCL8 were predicted target genes in tumor cells (**Figures 7A, B**). Secondly, plasma cells were also analyzed using receptors as markers. The plasma cells were found to be affected by CXCL8 secreted by the tumor cells, and the predicted target gene was CD24 (**Figures 7C, D**).

### Machine learning to optimize predictive models for prognosis

**Figure 8:**
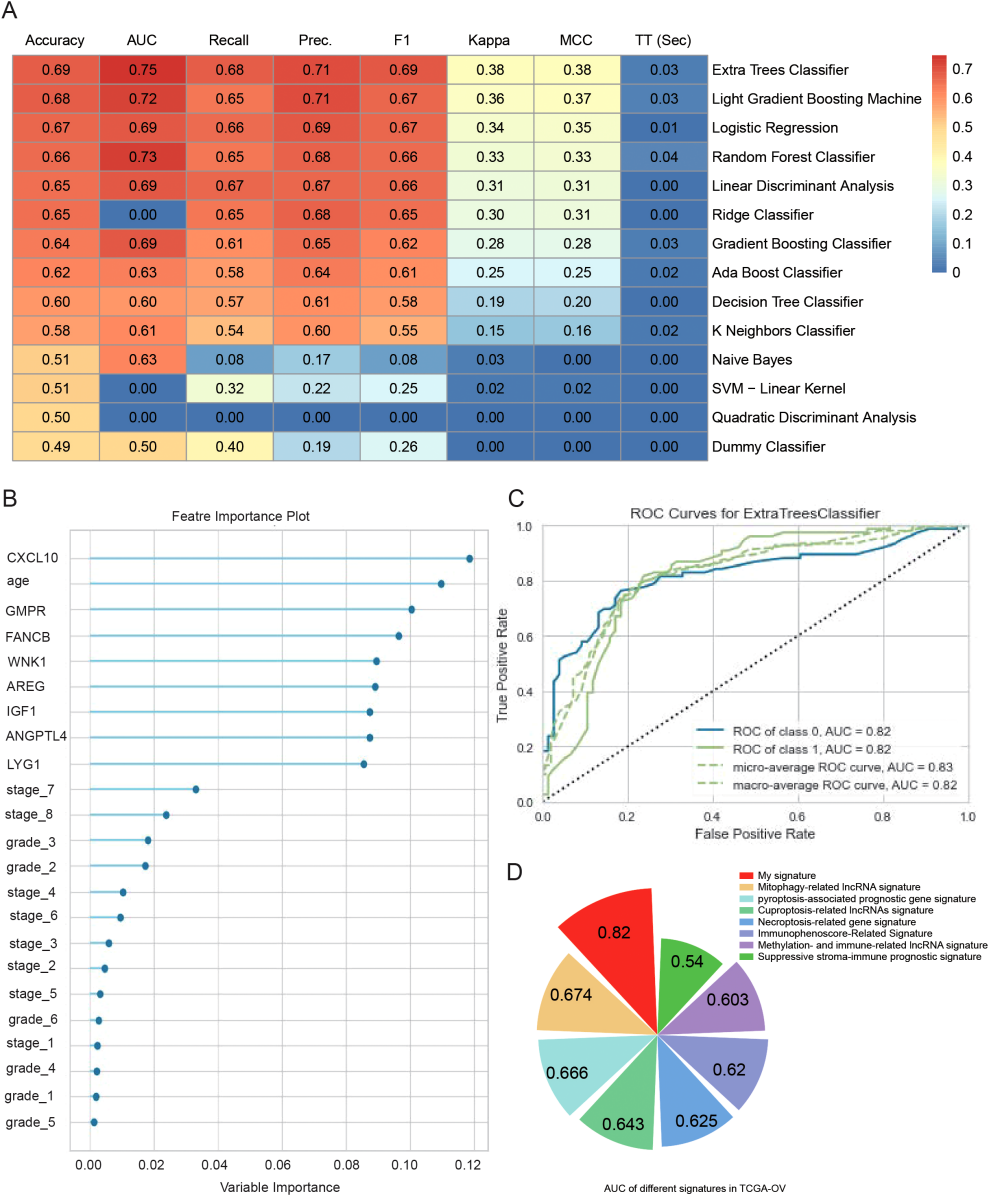
Machine learning to optimize predictive models for prognosis. **(A)** Heatmap comparison of multiple machine learning models using a TCGA-OV cohort. The heatmap displays the accuracy, area under the curve (AUC) value, recall, precision, F1 score, Kappa, and Matthews correlation coefficient (MCC) for each of the 14 classifiers tested. **(B)** Feature importance plots of the best model (Extra Trees Classifier) for predicting 5-year OS in TCGA-OV. **(C)** ROC curves of the best model (Extra Trees Classifier) for predicting 5-year OS in TCGA-OV. **(D)** Comparison of predictive models for prognosis in this paper and other literature.

A machine learning model based on the expression levels of eight prognostic genes combined with clinical features was developed to predict the five-year overall survival of ovarian cancer patients. To further improve prediction accuracy, we compare 14 machine learning algorithms and filter out the optimal prediction model: Extra Trees Classifier (**Figure 8A**). The optimal prediction model showed that all 8 genes had higher weights than tumor grade and FIGO stage (**Figure 8B**). The AUC of our predictive model for prognosis was 0.82 after optimization (**Figure 8C**). These data suggest that these 8 genes play an important role in predicting ovarian cancer prognosis and provide more prognostic value than traditional clinical information. The model was also validated by 2 external datasets (GSE140082 and GSE30161). The results showed that the model can effectively predict the prognosis of ovarian cancer patients (**Supplementary Figure S3**). A comparison between this model and other ovarian cancer prognostic models that have been previously described in the literature was conducted (**Figure 8D**). The models we developed were more accurate because of a higher AUC.

### Analysis of experimental and clinical data from clinical samples

**Figure 9:**
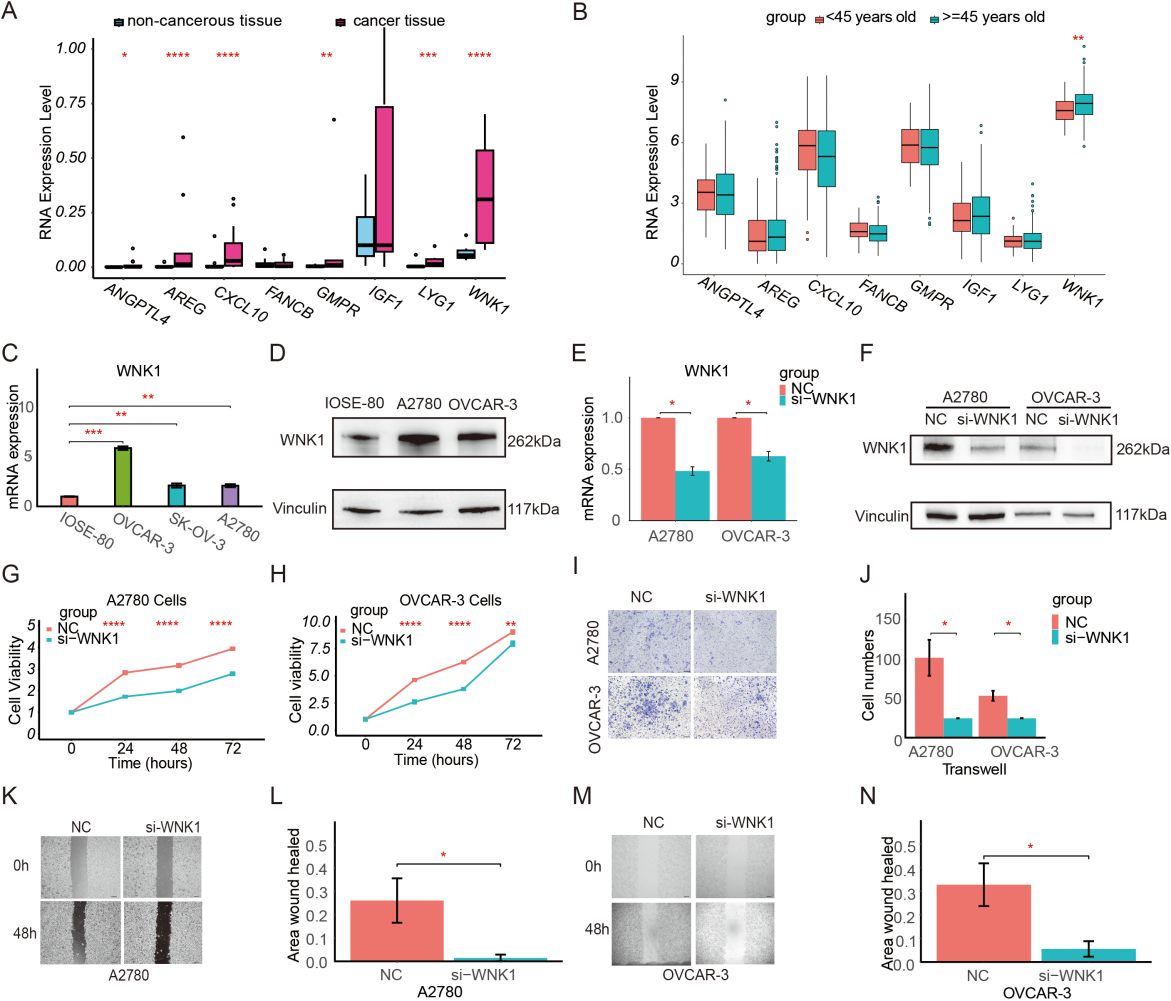
Expression level and function of WNK1 in ovarian cancer. **(A)** Validation of the differential expression of prognostic model genes at the RNA level by qRT-PCR in clinical patient tissues. **(B)** Comparison of the expression of model genes in patients in the premenopausal group (<45 years old) and the menopausal/postmenopausal group (>=45 years old) in the TCGA database. **(C)** Validation of the differential expression of WNK1 mRNA in IOSE-80, OVCAR-3, SK-OV-3, and A2780 cells by qRT-PCR. **(D)** Validation of the differential expression of WNK1 protein by Western blot. Vinculin was used as an internal loading control. **(E)** Measurement of the knockdown efficiency of WNK1 mRNA in ovarian cancer cells (A2780, OVCAR-3) by qRT-PCR. **(F)** Measurement of the knockdown efficiency of WNK1 protein in ovarian cancer cells (A2780, OVCAR-3) by Western blot. **(G)** Detection of the effect of WNK1 knockdown on A2780 cell viability using a CCK-8 assay. **(H)** Detection of the effect of WNK1 knockdown on OVCAR-3 cell viability using a CCK-8 assay. **(I, J)** Detection of the effect of WNK1 knockdown on ovarian cancer cell migration by Transwell migration assay (scale bar: 50 µm) **(K, L)**. Detection of the effect of WNK1 knockdown on A2780 cell migratory by wound-healing assay (scale bar: 200 µm). **(M, N)**. Detection of the effect of WNK1 knockdown on OVCAR-3 cell migratory by wound-healing assay (scale bar: 200 µm). ns, not significance; *p<0.05; **p<0.01; ***p<0.001; ****p<0.0001.

Ovarian samples were collected and the differential mRNA expression of CSOARG hub genes in ovarian non-malignant and malignant tumor tissues was examined. Our findings indicate that the majority of hub genes were overexpressed in malignant tumor samples in which *WNK1* was overexpressed most significantly detected by qRT-PCR (**Figure 9A**). The expression levels of six genes were found to be significantly elevated in ovarian cancer cells, except for FANCB and IGF1, which were not statistically significant. The data from the TCGA-OV cohort were divided into two groups based on an age-related to menopause: a premenopausal group (<45 years old) and a menopausal/postmenopausal group (>=45 years old) (**Figure 9B**). Our findings demonstrated a statistically significant overexpression of WNK1 in the menopausal/postmenopausal group. Further, qPCR and Western blot revealed that WNK1 was highly expressed in A2780 and OVCAR-3 ovarian cancer cells in comparison to IOSE-80 non-cancerous cells (**Figures 9C, D**). Given the significant overexpression of WNK1 in ovarian cancer, we proceeded to investigate the impact of WNK1 on ovarian cancer cell biological behavior. The *WNK1* gene was then knocked down in tumor cells using the siRNA transfection approach. The initial step was to ascertain the efficacy of si-WNK1 in knockdown in both OVCAR-3 and A2780 cells at RNA (**Figure 9E**) and protein levels (**Figure 9F**) by qRT-PCR and Western blot, respectively. The cell viability of ovarian cancer cells after the knockdown of WNK1 was decreased compared to the control group at 24, 48, and 72 h (**Figures 9G, H**). Next, the effect of WNK1 on cell migration was verified using a transwell migration assay and a wound-healing assay. The transwell migration assay demonstrated that the knockdown of WNK1 significantly reduced A2780 and OVCAR-3 cell migratory capacity compared to controls at 48 h (**Figures 9I, J**). The results of the wound-healing assay also confirmed that the knockdown of WNK1 decreased cell viability at 48 h (**Figures 9K–N**).

A risk score was calculated for each patient based on qRT-PCR values. This allowed for the classification of patients into high- and low-risk score groups. A comparative analysis of malignant tumors revealed no significant differences between high- and low-risk score groups concerning clinical characteristics (**Supplementary Table S5**). Nevertheless, the P-values for the difference in age between the high- and low-risk score groups were 0.08 and 0.15, respectively.

## Discussion

Ovarian cancer is the most lethal gynecologic malignancy, which is influenced by numerous factors. Tumor size and pathological grading are the most significant prognostic factors. Other factors, such as age, also have a substantial predictive impact on the prognosis of ovarian cancer. However, to date, there is no reliable method to predict the prognosis of ovarian cancer based on cellular senescence and ovarian aging-related genes.

The current study developed a predictive model of CSOARG for prognosis in ovarian cancer using eight key genes (*WNK1, ANGPTL4, AREG, IGF1, CXCL10, GMPR, FANCB, LYG1*). Patients were stratified into high- and low-risk score groups for analysis of the immune function and immunotherapy effects of 8 genes. The majority of these eight genes have been documented to be associated with tumors. CXCL10 is linked to the therapeutic effect of PD-1 and the inhibition of CXCL10 enhances the therapeutic effect of anti-PD-1 (31). IGF-1 is known to promote the invasive and EMT process of tumors as well as tumor resistance (32). ANGPTL4 promotes the proliferation and metastasis of tumors and the ability of angiogenesis (33). AREG hinders anti-tumor immunity, which is associated with stemness and chemoresistance in ovarian cancer (34). WNK1 promotes tumor migration and invasion in breast cancer (35). Inhibition of the WNK1-MEK5-ERK5 pathway can exert antiproliferative effects and enhance trametinib responsiveness in ovarian cancer (36). LYG1, FANCB, and GMPR are rarely reported in tumors and their functions need to be further investigated.

The present study found that CD4 memory T cell resting was elevated in the high-risk score group, indicating that CD4 memory T cell activation is diminished. It has been shown that resting CD4 memory cells exhibit diminished anti-tumor immunity relative to activated CD4 memory T cells (37). A focused analysis of immune checkpoints revealed a general down-regulation of HLA molecules in the high-risk score group, which may decrease the ability to recognize and present antigens in tumors and increase immune escape (38). Therefore, we conducted further analysis of the effect of immune profiles. The IPS score demonstrated that the high-risk score group expressed lower levels of immune checkpoint genes PD-1 and CTL-4, which resulted in a weaker capacity for antigen recognition. It has been shown that the IPS score is a superior predictor of response to anti-PD-1 and anti-CTLA-4 antibodies (25). Further analysis of the immune function of T cells was conducted to predict the effect of immunotherapy. The high-risk score group exhibited diminished T cell function, increased exclusion, and diminished tumor killing by T cells. T cells are the most common effector cells of immune checkpoint inhibitor therapy, thus inferring that immunotherapy was less effective in the high-risk score group. The recognition of antigens, the presentation of antigens to T cells, and the effector function of T cells are all diminished in the high-risk score group, indicating that immunotherapy is ineffective. Furthermore, we predicted differences in drug sensitivity between high- and low-risk score groups. The high-risk cohort exhibited higher IC_50_ values for drugs such as cisplatin, 5-FU, and camptothecin, which may result in a worse prognosis. It has been shown that cisplatin can cause tumor cell death by disrupting the DNA double-strand of the tumor (39). Our model predicts that the high-risk group has lower HRD and higher genomic stability. Tumor cells in the high-risk group are less likely to be killed by cisplatin. Additionally, some other drugs have been used as potential drugs for ovarian cancer treatment in animal models or clinical trials (40, 41).

Tumor cells exhibited the highest risk score, followed by immune cells and stromal cells which exhibited the lowest score. These data suggest that senescence-associated prognostic changes are mainly related to tumor cells and the immune environment. In the subgroup analysis, the proportion of plasma cells was significantly decreased in patients in the high-risk score group. Plasma cells may play an important role in tumor immunocytes. To further elucidate the mechanism underlying the reduction in plasma cells, we employed ligand/receptor analysis, which led to the preliminary inference that TGF-β affects tumor cells. Secretion of CXCL8 by tumor cells modulated the expression of plasma cell CD24, which ultimately attenuated the activation of B cells into plasma cells. These findings indicate that these molecules are associated with anti-tumor immunity. It has been hypothesized that cancer cells utilize CD24 molecules to evade detection and attack by the immune system. It has been reported that the expression of CD24 in mesenchymal stromal cells of human bone marrow is regulated by TGFβ3. In addition, it has been demonstrated that CD24 modulates the process of transforming growth factor β-induced epithelial-to-mesenchymal transition.

The senescence microenvironment, which is dominated by IL-6 and IL-8, promotes immune escape and metastasis of tumors (2). Additionally, tumor-associated macrophages (TAMs) can influence the senescence-associated tumor microenvironment by deactivating the function of T cells through TGF-β, which can influence the senescence-associated tumor microenvironment (42). It has been shown that TGF-β can control differentiation and proliferation and promote tumor progression, epithelial-mesenchymal transition (EMT), tumor invasiveness, and metastasis (43). Additionally, it has been demonstrated to weaken immune surveillance and promote immune escape (44). Inhibiting TGF-β enhances the efficacy of chemotherapy and synergistic immune checkpoint therapy (45, 46). It has been reported that CXCL8 (IL8) promotes tumor proliferation, migration, and invasion and is an important component of senescent secretory proteins (47). High expression of IL8 is associated with poor clinical prognosis and poor immune checkpoint efficacy (48). CD24 promotes tumor migration and inhibits anti-tumor responses associated with immune checkpoint therapy (49). Meanwhile, CD24 expression is associated with B-cell maturation (50).

Although numerous prognostic models exist for predicting the prognosis of ovarian cancer, there are currently no prognostic models derived from ovarian aging and senescence-related genes. Other prediction models such as gene labeling utilize factors such as necroptosis, cellular pyroptosis, and cuproptosis (11–13). However, these models lack sufficient accuracy. The proposed prediction model not only considers the tumor prognosis from the perspective of aging but also employs a more effective methodology to enhance the prediction accuracy. The AUC value of 0.82 in our CSOARG model was superior to that of the general prediction models. Our model can facilitate more precise assessments of patient status and the implementation of more precise treatments.

The current findings indicate that the majority of hub genes are upregulated in malignant tumors. The detection of upregulated genes in the clinical setting is a relatively straightforward process, and these genes can be utilized as prognostic markers. WNK1 is the gene with the highest coefficient among the 8 genes used to construct the prognostic model. In addition, WNK1 expression was found to be the highest one among 8 genes in clinical sample validation. Furthermore, our findings demonstrated that WNK 1, the molecule that differs most markedly in patients with non-malignant and malignant tumors, promoted cell proliferation and migration and thus acts as an oncogene. WNK1 can promote tumor proliferation through the WNT/β-Catenin signaling pathway (51). We found that the knockdown of WNK1 inhibited ovarian cancer cell migration. It has been shown that WNK1 activates NFAT transcription factors and promotes cell migration in clear cell renal cell carcinoma (ccRCC) (52). A recent study has revealed a positive correlation between the activation of the WNK1-OSR1-NKCC1 axis and the invasiveness of liver cancer cell lines (35). It has been demonstrated that inhibition of WNK1 by a specific inhibitor WNK463 effectively reduces the expression level of the senescence marker β-galactosidase and affects an oncogene *MYC* transcription, in tumor cells (53, 54). In addition, age-related hearing loss is associated with WNK1 (55). Furthermore, WNK1 has a pathophysiological role and influences the efficacy of trametinib in ovarian cancer (36). It has also been shown that WNK1 increased cisplatin resistance in ovarian cancer cells (56). These findings suggest that WNK1 is closely associated with senescence and targeting WNK1 may be a viable strategy to impede tumor senescence.

The limitations of this study still exist. First, more experiments are needed to investigate specific molecular regulatory mechanisms in the future. Second, all of the aforementioned data are retrospective analyses from public databases. Third, the validation sample size is relatively small. To verify the accuracy of the model and the clinical value of precise treatment, prospective cohorts are needed.

In conclusion, the present findings demonstrate that a novel CSOARG model can effectively predict the prognosis and therapeutical responses of patients with ovarian cancer, which may assist clinicians in implementing better practices.

## Data Availability

The original contributions presented in the study are included in the article/**Supplementary Material**. Further inquiries can be directed to the corresponding author.
