# Supplementary material for: Development of CSOARG: a single-cell and multi-omics-based machine learning model for ovarian cancer prognosis and drug response prediction
Source: Front Oncol. 2025 May 29;15:1592426. doi: 10.3389/fonc.2025.1592426 (PMC12159008; doi:10.3389/fonc.2025.1592426)
Supplement: Supplementary file 1 [file DataSheet1.pdf]

Supplementary materials:

Table S1 Ovarian aging-related genes.

Ovarian aging-related genes						
ABHD12	*CCNT2*	*FAM172BP*	*INO80*	*PARL*	*RPA1*	*TP53BP1*
ACACA	*CCT6A*	*FAM208B*	*IPO7*	*PELO*	*RPAIN*	*TP63*
ACSBG1	*CDIP1*	*FAM83F*	*IPO8*	*PEX5*	*RREB1*	*TRA2A*
ACSL1	*CDK2AP1*	*FANCA*	*ITIH3*	*PFKM*	*RTEL1*	*TRIM33*
ACSL3	*CHAMP1*	*FANCB*	*KCNQ1*	*PIWIL1*	*RUNX1*	*TSC22D2*
ADNP	*CHD7*	*FANCI*	*KDELR3*	*PMF1*	*SBNO1*	*U2SURP*
ADSS	*CHEK2*	*FANCM*	*KLF10*	*PNKP*	*SCAP*	*UBE2L3*
AFF3	*CIT*	*FBXL17*	*KNL1*	*PNP*	*SCCPDH*	*UBXN2A*
AL162431.1	*CNNM2*	*FGFRL1*	*KPNA3*	*POLG*	*SERAC1*	*UFD1L*
ANAPC4	*CPEB1*	*FIGNL1*	*L3MBTL3*	*PPARG*	*SH3PXD2B*	*UIMC1*
ANKRD30A	*CPEB4*	*FLJ26245*	*LBR*	*PPIH*	*SIRT1*	*UPRT*
APOLD1	*CPNE1*	*FOXO1*	*LHCGR*	*PPM1B*	*SLC25A12*	*UQCRC1*
APTX	*CTD-2085J24.4*	*FSHB*	*LINC00824*	*PPM1F*	*SLC9A8*	*USP3*
ARHGEF18	*DCAF4L1*	*FTO*	*LRRC32*	*PPP1CB*	*SLCO4A1*	*USP34*
ARID3A	*DDIT4L*	*GAB2*	*LYG1*	*PPP2R3A*	*SLMAP*	*USP7*
ARID3B	*DDX5*	*GBAS*	*MAFTRR*	*PPP5C*	*SNTA1*	*USPL1*
ATCAY	*DEFB125*	*GCKR*	*MAP2K2*	*PQLC1*	*SOD2*	*WASIR2*
ATXN8OS	*DENND1A*	*GEMIN5*	*MBTPS2*	*PRIM1*	*SPATA2*	*WDR12*
BAG5	*DEPTOR*	*GJA1*	*MCM10*	*PRKDC*	*SPRTN*	*WNK1*
BCL2	*DIDO1*	*GK*	*MCM8*	*PRR3*	*SPRY4*	*WRB*
BCL2L11	*DMRT1*	*GMPR*	*MICA*	*PRRC2A*	*SPTSSA*	*WT1*
BIRC6	*DNA2*	*GNAS*	*MIR3977*	*PTPRJ*	*SRP9*	*WWOX*
BLK	*DNAJC11*	*GORASP2*	*MLN*	*PVT1*	*SRPK1*	*YBX2*
BLNK	*DNAJC7*	*GPCPD1*	*MON1A*	*R3HDM2*	*STAG3*	*YBX3*
BMP4	*DNER*	*GSPT1*	*MRPS31*	*RAD18*	*STON1-GTF2A1L*	*YDJC*
BMPR1B	*DPPA3*	*GSTM4*	*MSH6*	*RAD51*	*SV2A*	*YY2*
BOD1L1	*EAPP*	*HCAR2*	*MSI2*	*RAD54L*	*SYCP2L*	*ZAR1*
BRCA1	*EBF3*	*HEATR3*	*NADK2*	*RASAL2*	*TBC1D1*	*ZCCHC2*
BRCA2	*EFHC2*	*HELB*	*NAMPT*	*RBBP8*	*TDRD3*	*ZCCHC8*
BRD3	*EIF4E*	*HELQ*	*NDUFV3*	*RBM25*	*TFAP2C*	*ZFP42*
BRE	*EIF4EBP1*	*HIST1H2BJ*	*NLRP11*	*RBM26*	*TGFBR2*	*ZFPM1*
BTBD1	*ELAVL2*	*HLA-B*	*NLRP4*	*RBM46*	*TIPARP*	*ZNF208*
C11orf58	*ENTPD1-AS1*	*HLA-DQA1*	*NMRK2*	*REV3L*	*TLK1*	*ZNF275*
C16orf72	*ERBB2*	*HLA-E*	*NOBOX*	*RHBDL2*	*TMEM120B*	*ZNF518A*
C17orf80	*ERLIN1*	*HOXD4*	*NSUN4*	*RIBC2*	*TMEM169*	*ZNF638*
C19orf57	*ESRP1*	*IGF1*	*NUP62*	*RIF1*	*TMEM86B*	*ZNF704*
C1orf112	*ESYT2*	*IGFBP1*	*OOEP*	*RNF220*	*TNPO3*	*ZNF728*
CALCR	*ETAA1*	*IKZF2*	*OPA1*	*RNF44*	*TNRC18*	*ZNF91*
CARF	*ETS1*	*ING4*	*OR5V1*	*RP11-158M2.5*	*TOP3A*	*ZRANB1*
CASC22	*EXO1*	*INHBB*	*PAPD7*	*RP5-864K19.4*	*TOP3B*	*ZXDC*
CBFA2T3	*FAAP24*	*INHBC*				

Note: The data in a geneset were extracted from the study of Ruth, K.S., et al [1].

Table S2 Cellular senescence genes from SenMayo geneset.

Cellular senescence genes					
ACVR1B	*CCL7*	*EGFR*	*IGFBP6*	*MMP1*	*SCAMP4*
ANG	*CCL8*	*EREG*	*IGFBP7*	*MMP10*	*SELPLG*
ANGPT1	*CD55*	*ESM1*	*IL10*	*MMP12*	*SEMA3F*
ANGPTL4	*CD9*	*ETS2*	*IL13*	*MMP13*	*SERPINB4*
AREG	*CSF1*	*FAS*	*IL15*	*MMP14*	*SERPINE1*
AXL	*CSF2*	*FGF1*	*IL18*	*MMP2*	*SERPINE2*
BEX3	*CSF2RB*	*FGF2*	*IL1A*	*MMP3*	*SPP1*
BMP2	*CST4*	*FGF7*	*IL1B*	*MMP9*	*SPX*
BMP6	*CTNNB1*	*GDF15*	*IL2*	*NAP1L4*	*TIMP2*
C3	*CTSB*	*GEM*	*IL32*	*NRG1*	*TNF*
CCL1	*CXCL1*	*GMFG*	*IL6*	*PAPPA*	*TNFRSF10C*
CCL13	*CXCL10*	*HGF*	*IL6ST*	*PECAM1*	*TNFRSF11B*
CCL16	*CXCL12*	*HMGB1*	*IL7*	*PGF*	*TNFRSF1A*
CCL2	*CXCL16*	*ICAM1*	*INHA*	*PIGF*	*TNFRSF1B*
CCL20	*CXCL2*	*ICAM3*	*IQGAP2*	*PLAT*	*TUBGCP2*
CCL24	*CXCL3*	*IGF1*	*ITGA2*	*PLAU*	*VEGFA*
CCL26	*CXCL8*	*IGFBP1*	*ITPKA*	*PLAUR*	*VEGFC*
CCL3	*CXCR2*	*IGFBP2*	*JUN*	*PTBP1*	*VGF*
CCL3L1	*DKK1*	*IGFBP3*	*KITLG*	*PTGER2*	*WNT16*
CCL4	*EDN1*	*IGFBP4*	*LCP1*	*PTGES*	*WNT2*
CCL5	*EGF*	*IGFBP5*	*MIF*	*RPS6KA5*	

Note: The cellular senescence geneset, named SenMayo, was identified from the study of Saul, D., et al [2].

Table S3 Coefficients of genes in the CSOARG model.

Gene	Coef
WNK1	0.120974
ANGPTL4	0.099811
AREG	0.087816
IGF1	0.052135
CXCL10	-0.06992
GMPR	-0.15972
FANCB	-0.20232
LYG1	-0.23654

Note: The coefficients (Coef) were obtained by a LASSO regression (https://glmnet.stanford.edu/articles/glmnet.html).

Table S4 Primer sequences are used in this article.

Primer	Sequence（5‘-3’）	GenBank ID	Position
**ACTB**		NM_001101	
Forward	CATGTACGTTGCTATCCAGGC		393-413
Reverse	CTCCTTAATGTCACGCACGAT		642-622
**ANGPTL4**		NM_001039667	
Forward	GTCCACCGACCTCCCGTTA		255-273
Reverse	CCTCATGGTCTAGGTGCTTGT		466-446
**AREG**		NM_001657	
Forward	GTGGTGCTGTCGCTCTTGATA		31-51
Reverse	CCCCAGAAAATGGTTCACGCT		127-107
**CXCL10**		NM_001565	
Forward	GTGGCATTCAAGGAGTACCTC		50-70
Reverse	TGATGGCCTTCGATTCTGGATT		247-226
**FANCB**		NM_001018113	
Forward	ATGAAGGATGGCCTAAGGGTC		388-408
Reverse	ACACACTAACAACTTTGCCAGT		490-469
**GMPR**		NM_006877	
Forward	CTCAAGCTCGACTTCAAGGATG		22-43
Reverse	GGGAATCCCTGAGTAGGTCTG		147-127
**IGF1**		NM_001111283	
Forward	GCTCTTCAGTTCGTGTGTGGA		181-201
Reverse	GCCTCCTTAGATCACAGCTCC		313-293
**LYG1**		NM_174898	
Forward	TGGAGCATCTTGTGGGATTGG		102-122
Reverse	TGGCATGTCTATTTCAGCCAG		189-169
**WNK1**		NM_014823	
Forward	GCCGTCAGATCCTTAAAGGTC		983-1003
Reverse	CCAGTAGGGCCGGTGATAA		1085-1067

The sequences were identified from the GeneBank at https://www.ncbi.nlm.nih.ggovv//genbank.

Table S5 Risk score and clinical data analysis for patients with ovarian cancer.

Characteristics	Low-risk (n=6)	High-risk (n=5)	P-value
Age (mean±SD)	54.8±10.5	64.6±8.4	0.08 [a]
Menopause			0.15 [b]
Yes	4	5	
No	2	0	
CA125, median (IQR)	2520.05 (604.725,2932.375)	5134 (116,5134)	0.41 [a]
CA153, median (IQR)	44.9 (17.71,82.875)	161.4 (11.7,279.2)	0.66 [a]
FIGO stage (2 missing)			0.34 [a]
I	0	0	
II	0	0	
III	4	2	
IV	1	2	

Note: Samples from 11 patients were collected. Based on the risk scores, 6 samples were in the low-risk group and 5 samples were in the high-risk group. a: Wilcoxon Mann-Whitney test; b: Chi-square test; n, number of cases; SD, standard deviation; IQR, interquartile range; FIGO, International Federation of Gynecology and Obstetrics.

References

1. Ruth, K.S., et al., Genetic insights into biological mechanisms governing human ovarian ageing. Nature, 2021. 596(7872): p. 393-397.
2. Saul, D., et al., A new gene set identifies senescent cells and predicts senescence-associated pathways across tissues. Nat Commun, 2022. 13(1): p. 4827.
