# Supplementary material for: Development of CSOARG: a single-cell and multi-omics-based machine learning model for ovarian cancer prognosis and drug response prediction
Source: Front Oncol. 2025 May 29;15:1592426. doi: 10.3389/fonc.2025.1592426 (PMC12159008; doi:10.3389/fonc.2025.1592426)
Supplement: Supplementary file 2 [file DataSheet2.pdf]

Supplementary Figures

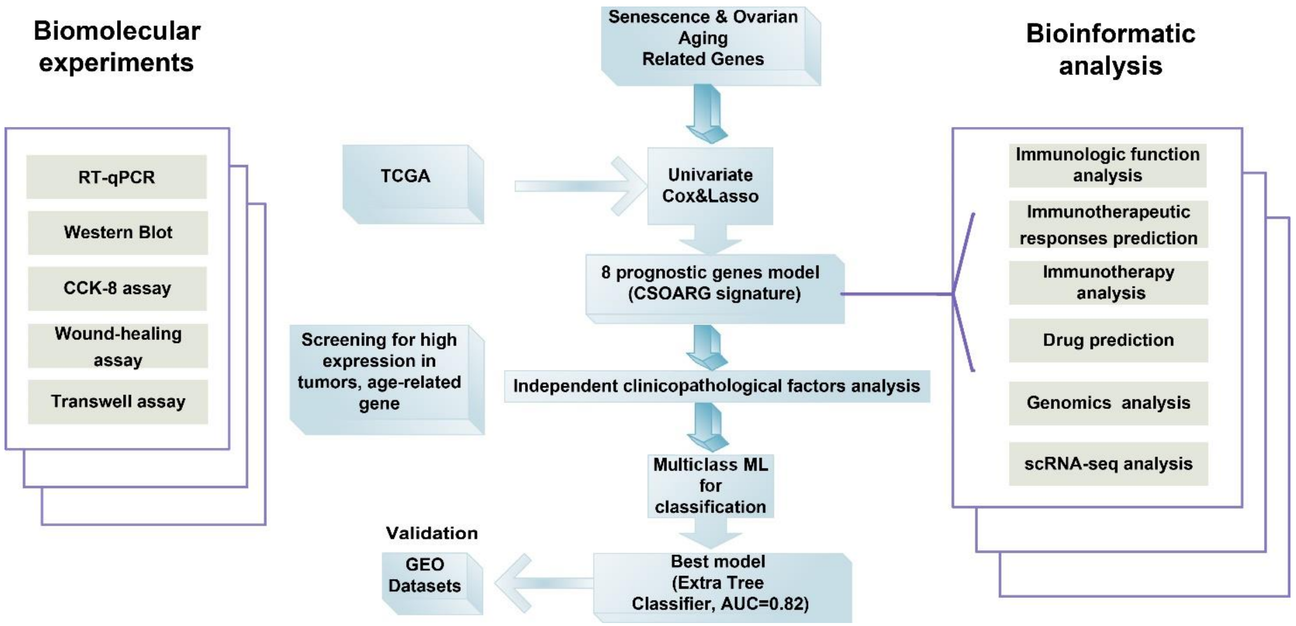

Figure S1. Workflow of constructing and analyzing the predictive model of CSOARG for the prognosis of patients with ovarian cancer.

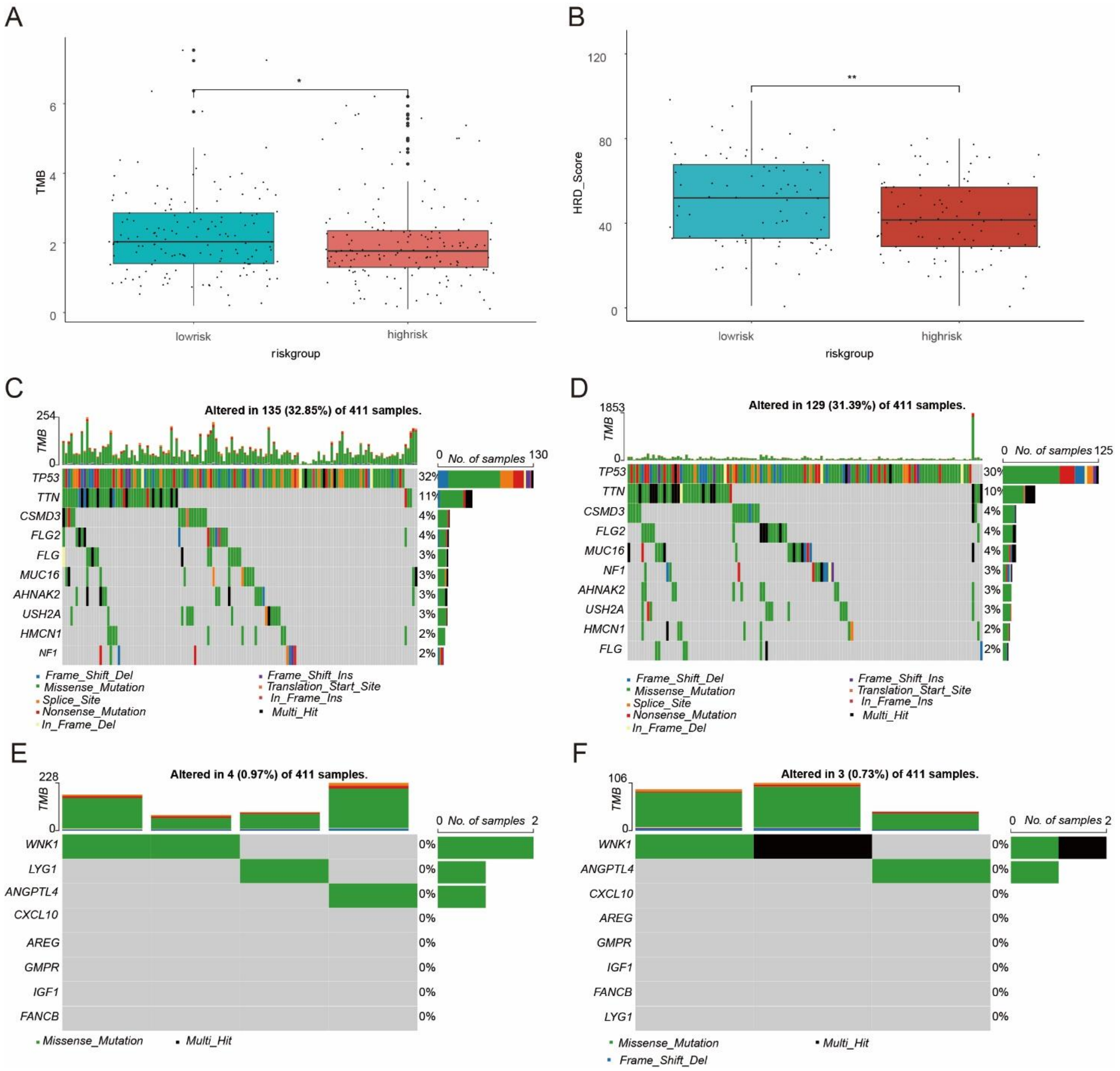

Figure S2. Genomic instability analysis. (A) The boxplot shows the levels of tumor mutation burden in patients between the low-risk and high-risk score groups in the TCGA-OV cohort. (B) The boxplot shows the levels of Homologous recombination deficiency (HRD) scores in patients between high- and low-risk groups in the TCGA-OV cohort. (C, D) The oncoplot shows the landscape genome changes in patients with different CSOARG subtypes in the low-risk group (C) and the high-risk group (D). (E, F) The oncoplot shows the model-construction gene changes in patients with different SIPS subtypes in the low-risk group (E) and the high-risk group (F).

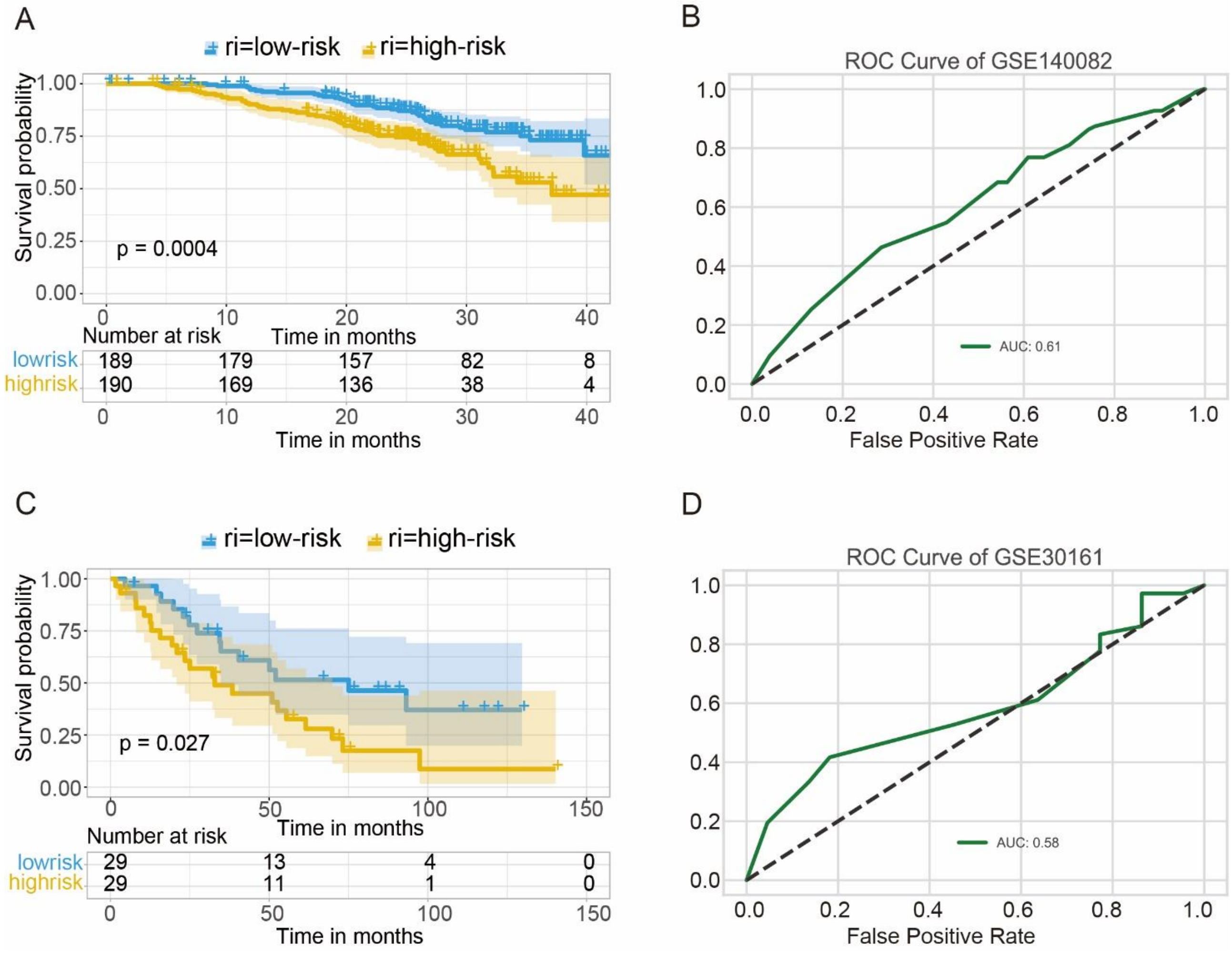

Figure S3. External data validation. (A) Kaplan-Meier plot of GSE140082 dataset. (B) Area under receiver-operating characteristic (ROC) curve of the GSE140082 dataset. (C) Kaplan-Meier plot of the GSE30161 dataset. (D) Area under ROC curve of GSE30161 dataset.
